# Predictors and Prognostic Impact of Perioperative Hypotension During Transcatheter Aortic Valve Implantation: The Role of Diabetes Mellitus and Left Ventricular Dysfunction

**DOI:** 10.3390/jcdd12100398

**Published:** 2025-10-09

**Authors:** Zeynep Ece Demirbaş, Şahin Yılmaz, Fatma Can, Gönül Zeren, Can Yücel Karabay

**Affiliations:** 1 Department of Internal Medicine, Dr. Siyami Ersek Thoracic and Cardiovascular Surgery Training and Research Hospital, Istanbul 34668, Turkey; 2 Department of Anesthesiology, Dr. Siyami Ersek Thoracic and Cardiovascular Surgery Training and Research Hospital, Istanbul 34668, Turkey; 3 Department of Cardiology, Dr. Siyami Ersek Thoracic and Cardiovascular Surgery Training and Research Hospital, Istanbul 34668, Turkey

**Keywords:** transcatheter aortic valve implantation (TAVI), perioperative hypotension, diabetes mellitus, left ventricular ejection fraction, anesthesia management, mortality

## Abstract

Background: Perioperative hypotension is a frequent but underrecognized complication during transcatheter aortic valve implantation (TAVI). Although reduced left ventricular ejection fraction (EF) and low baseline blood pressure have been linked to hemodynamic instability, the role of metabolic comorbidities and procedural factors remains less well established. Methods: We retrospectively analyzed 123 patients who underwent transfemoral TAVI between June 2016 and June 2022. Perioperative hypotension was defined as a sustained systolic blood pressure < 90 mmHg or ≥30% reduction from baseline for at least 5 min. Clinical, laboratory, and procedural predictors were assessed using multivariate logistic regression, and model performance was evaluated by ROC curve analysis. Results: Perioperative hypotension occurred in 57% of patients. Independent predictors were diabetes mellitus (OR 2.79, 95% CI 1.03–7.56, *p* = 0.044), reduced EF (<50%) (OR 2.87, 95% CI 1.13–7.31, *p* = 0.027), lower baseline diastolic blood pressure (OR 0.935 per mmHg, 95% CI 0.893–0.978, *p* = 0.004), and longer procedural duration (OR 1.038 per minute, 95% CI 1.001–1.076, *p* = 0.044). The predictive model demonstrated good calibration and discrimination (AUC 0.844). Patients with hypotension had significantly higher in-hospital mortality (12.9% vs. 1.9%, *p* = 0.027) and longer ICU stay. An exploratory finding suggested less frequent use of sugammadex among hypotensive patients (11.4% vs. 32.1%, *p* = 0.005). Conclusions: Perioperative hypotension is common during TAVI and strongly associated with early mortality. Our study uniquely identifies diabetes mellitus as an independent predictor, alongside ventricular dysfunction, baseline blood pressure, and procedural duration. These findings suggest that careful preprocedural risk stratification, hemodynamic vigilance, and optimization of anesthetic management may improve outcomes in vulnerable patients.

## 1. Introduction

Transcatheter aortic valve implantation (TAVI) has emerged as a transformative treatment modality for patients with severe aortic stenosis who are at high or prohibitive surgical risk [1]. Over the past decade, the procedure has demonstrated significant improvements in survival and quality of life, even in elderly and frail populations [2,3]. However, the success of TAVI is closely tied to the meticulous management of intraoperative and perioperative hemodynamic parameters [4,5].

Among these, perioperative hypotension is a frequent yet underrecognized complication that may compromise organ perfusion, prolong recovery, and contribute to adverse outcomes [6,7]. The transient but significant blood pressure fluctuations observed during TAVI are influenced by multiple factors, including rapid ventricular pacing, anesthetic agents, baseline cardiovascular reserve, and vascular access techniques [8,9].

While the association between perioperative hypotension and poor outcomes is well established in cardiac surgery and other high-risk interventions, its prognostic significance in the context of TAVI remains less clearly defined. Recent studies have suggested that even short episodes of hypotension during the TAVI procedure may be associated with an increased risk of vascular complications, acute kidney injury, prolonged intensive care unit stay, and mortality [6,10]. However, the specific patient and procedural characteristics that predispose individuals to hypotension—and the magnitude of its impact on postprocedural outcomes—are not yet fully understood.

Given the growing number of TAVI procedures worldwide and the expanding indications to include intermediate- and low-risk patients, understanding the determinants and consequences of perioperative hypotension is of critical importance. Identifying modifiable risk factors could lead to targeted preventive strategies and ultimately improve procedural safety.

In this study, we aimed to investigate the clinical, laboratory, and procedural predictors of perioperative hypotension in patients undergoing TAVI and to evaluate its relationship with early postoperative outcomes, including in-hospital mortality.

## 2. Materials and Methods

This retrospective observational study was conducted at Dr. Siyami Ersek Thoracic and Cardiovascular Surgery Training and Research Hospital in Istanbul, Turkey. The study included patients who underwent transcatheter aortic valve implantation (TAVI) between June 2016 and June 2022. Patients who underwent elective transfemoral TAVI procedures during the study period were retrospectively identified from the hospital’s electronic medical records. Inclusion criteria were: age ≥ 18 years, complete perioperative hemodynamic data, and availability of preoperative laboratory results. Patients with missing key data (e.g., anesthesia records, hemodynamic monitoring, or laboratory parameters) or those who underwent emergency or salvage procedures were excluded from the study. Only elective transfemoral TAVI procedures were included. A total of 257 consecutive patients undergoing transfemoral TAVI were screened. After exclusion of patients with missing intraoperative hemodynamic data and/or missing preoperative biochemical values, 123 patients remained for the final analysis.

### 2.1. Data Collection

Demographic information (age, sex, BMI), comorbidities like diabetes mellitus, hypertension, coronary artery disease (CAD), chronic kidney disease (CKD), prior stroke, smoking history; echocardiographic findings like left ventricular ejection fraction (EF), and medication history, including use of beta-blockers, angiotensin-converting enzyme inhibitors (ACE-Is), angiotensin II receptor blockers (ARBs), non-vitamin K antagonist oral anticoagulants (NOACs), warfarin, adenosine diphosphate (ADP) receptor blockers, statins and diuretics were recorded. Preoperative systolic and diastolic blood pressure values (SBP, DSP) and heart rate (HR) were noted. Laboratory data included serum albumin, lipid profile, hemoglobin, hematocrit, platelet count, liver function tests (ALT, AST, GGT), and total bilirubin.

Procedural variables such as total procedure duration, total anesthetic time, anesthetic agents used (midazolam, propofol, dexmedetomidine, rocuronium, sugammadex), were noted. Clinical outcomes were defined and categorized according to the Valve Academic Research Consortium-2 (VARC-2) criteria, including device success, bleeding, vascular complications, stroke/TIA, myocardial infarction, acute kidney injury, permanent pacemaker implantation, and mortality [11].

### 2.2. Outcome Definition

Perioperative hypotension was defined as a sustained drop in systolic blood pressure < 90 mmHg or a ≥30% decrease from baseline for at least 5 consecutive minutes during the TAVI procedure, based on anesthetic records and intraoperative monitoring data.

### 2.3. Statistical Analysis

Continuous variables were tested for normality using the Kolmogorov–Smirnov test and are presented as mean ± standard deviation or median (min-max), as appropriate. Categorical variables are expressed as frequencies and percentages. Comparisons between hypotension and non-hypotension groups were performed using the Chi-square or Fisher’s exact test for categorical variables and Student’s *t*-test or Mann–Whitney U test for continuous variables.

Univariate logistic regression was used to identify potential predictors of perioperative hypotension. Variables with a *p*-value < 0.10 or deemed clinically relevant were entered into a multivariate logistic regression model using the backward likelihood ratio method. Model performance was evaluated using Nagelkerke R^2^, the Hosmer–Lemeshow goodness-of-fit test, and the area under the receiver operating characteristic (ROC) curve. To reduce the risk of overfitting, internal validation was performed using bootstrap resampling with 1000 iterations. Odds ratios (OR) with 95% bias-corrected accelerated confidence intervals were reported. A *p*-value < 0.05 was considered statistically significant. All analyses were performed using IBM SPSS Statistics, version 29 (IBM Corp., Armonk, NY, USA).

## 3. Results

A total of 123 patients who underwent TAVI were included in the study. Perioperative hypotension occurred in 70 patients (56.9%), while 53 patients (43.1%) remained hemodynamically stable during the procedure.

As shown in Table 1, the median age was similar between the hypotension and non-hypotension groups (81 vs. 80 years, *p* = 0.458), and no significant sex difference was observed (female: 47.1% vs. 50.9%, *p* = 0.676). The prevalence of coronary artery disease was comparable between groups (60.3% vs. 63.5%, *p* = 0.724). However, diabetes mellitus was more frequent among patients who developed hypotension (44.6% vs. 26.9%, *p* = 0.049). A markedly higher proportion of patients with low EF (<50%) experienced hypotension (61.8% vs. 31.4%, *p* = 0.001). Additionally, the mean EF was lower in patients with hypotension (50% vs. 55%, *p* = 0.001).

Baseline hemodynamics and outcomes of the patients are summarized in Table 2. Median preoperative SBP and DBP were significantly lower in the hypotension group (SBP: 129 vs. 150 mmHg, *p* < 0.001; DBP: 58.3 ± 12.5 vs. 66.3 ± 8.8 mmHg, *p* < 0.001) as expected.

Regarding procedural parameters, the median procedure time was significantly longer in the hypotension group (70 vs. 60 min, *p* = 0.005), and total anesthetic time was also higher (80 vs. 75 min, *p* = 0.032). No significant differences in procedure duration were observed between patients with and without diabetes mellitus or low EF, and baseline systolic and diastolic blood pressures were not correlated with procedure time (all *p* > 0.1). Analysis of VARC-2 defined outcomes demonstrated that device success was significantly lower in the hypotension group compared with those without hypotension (12.9% vs. 1.9%, *p* = 0.027). No life-threatening or disabling bleeding events occurred in either group, although median bleeding volume was higher among hypotensive patients (250 [150–350] vs. 200 [100–350] mL, p = 0.037). Major vascular complications were also more frequent in hypotensive patients (15.7% vs. 1.9%, p = 0.010) and hypotension was markedly more frequent among patients who experienced major vascular complications vice versa (11/12 [91.7%] vs. 59/111 [53.2%]; *p* = 0.012). There were no significant between-group differences in the rates of acute kidney injury (4.3% vs. 0%), stroke/TIA (2.9% vs. 0%), myocardial infarction (1.6% vs. 0%), arrhythmia (35.7% vs. 35.8%), or new permanent pacemaker implantation (34.3% vs. 28.3%). However, hypotension was associated with longer ICU stay (up to 31 days vs. 2 days, *p* = 0.032) and a higher in-hospital or 30-day mortality (12.9% vs. 1.9%, *p* = 0.027).

Laboratory results are summarized in Table 3. While most laboratory parameters were comparable between groups, patients with hypotension had significantly higher median triglyceride levels (116 vs. 87 mg/dL, *p* = 0.016). No significant differences were observed in hemoglobin, hematocrit, ALT, AST, GGT, or bilirubin levels.

As presented in Table 4, anesthetic medication profiles differed slightly between groups. All patients received propofol, while low-dose midazolam (1–2 mg) tended to be more common in the hypotension group (88.6% vs. 75.5%, *p* = 0.050), representing a borderline difference. Notably, sugammadex (Bridion) was used significantly less frequently in the hypotension group (11.4% vs. 32.1%, *p* = 0.005), suggesting a potential protective effect.

Multivariate logistic regression analysis was performed using variables with *p* < 0.10 in univariate analysis, as well as those with clinical relevance. As it is given after the procedure, sugammedex use was not included in logistic regression. As shown in Table 5 four factors remained independently associated with perioperative hypotension: diabetes mellitus (OR: 2.787, 95% CI: 1.027–7.561, *p* = 0.044), low EF (OR: 2.870, 95% CI: 1.126–7.313, *p* = 0.027), lower preoperative DBP (OR: 0.935, 95% CI: 0.893–0.978, *p* = 0.004) and longer procedure duration (OR: 1.038 per minute, 95% CI: 1.001–1.076, *p* = 0.044), and. The final regression model demonstrated good calibration (Hosmer–Lemeshow χ^2^ = 12.772, df = 7, *p* = 0.078) and explained 36.2% of the variance in hypotension occurrence (Nagelkerke R^2^ = 0.362). In multivariable logistic regression, diabetes mellitus (*p* = 0.036), reduced EF (*p* = 0.016), and lower baseline diastolic blood pressure (*p* = 0.002) emerged as independent predictors of perioperative hypotension, while procedure duration lost statistical significance after bootstrap resampling (*p* = 0.114). Bootstrap internal validation (1000 resamples) confirmed the robustness of the associations for diabetes, reduced EF, and diastolic blood pressure.

The final model demonstrated acceptable goodness-of-fit (Hosmer–Lemeshow test: χ^2^ = 12.772, df = 7, *p* = 0.078) and explained 36.2% of the variance (Nagelkerke R^2^ = 0.362). Bootstrap results are based on 1000 resamples (bias-corrected accelerated method).

**Figure 1:**
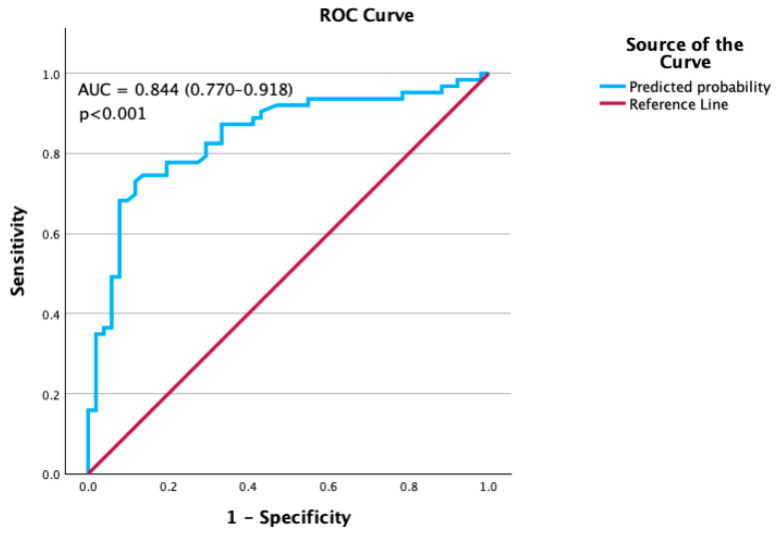
Receiver Operating Characteristic (ROC) curve for the prediction model.

Finally, the discriminative ability of the model was assessed using ROC curve analysis. The area under the curve (AUC) was 0.844 (95% CI: 0.770–0.918, *p* < 0.001), indicating good predictive accuracy (Figure 1). However, AUC values are based on the original model; bootstrap validation showed that procedure time was not a stable predictor, and thus the AUC may overestimate true discriminatory ability.

## 4. Discussion

In this single-center retrospective analysis of 123 patients undergoing transfemoral TAVI, perioperative hypotension occurred in 57% of the cohort. Importantly, hypotension was independently predicted by diabetes mellitus, reduced ejection fraction (<50%), lower baseline diastolic blood pressure, and longer procedural duration. To our knowledge, this is among the few studies to identify diabetes mellitus as an independent predictor of perioperative hypotension in the TAVI setting, and it provides additional insight into the interaction between preprocedural hemodynamics, procedural complexity, and outcomes.

The incidence of hypotension in our series is 56.9%. When compared with the recent study by Ni et al., who reported a post-induction hypotension incidence of approximately 32% in 643 TAVR patients using MAP-based definitions, the incidence in our cohort was substantially higher [7]. Several factors may explain this discrepancy. First, our population had a higher prevalence of reduced ejection fraction and diabetes mellitus, both of which are known risk factors for hemodynamic instability. Second, the majority of our procedures were performed under sedation with propofol and midazolam, agents that may predispose to hypotension, whereas dexmedetomidine was more commonly used in the Ni et al. cohort. Finally, our stricter definition based on systolic blood pressure (<90 mmHg or ≥20% decrease lasting ≥5 min) and continuous invasive monitoring may have led to more sensitive detection of clinically relevant hypotensive episodes. Together, these differences in patient profile, anesthetic protocols, and definitions likely account for the higher incidence observed in our study.

When compared with the pooled outcomes reported by Généreux et al. in a meta-analysis of 3519 patients using VARC definitions, our results showed broadly consistent event rates but with some notable deviations. Device success in our cohort (91.9%) matched the meta-analytic estimate of 92.1%, although it was significantly lower among hypotensive patients. Thirty-day mortality was 8.1%, closely aligned with the reported 7.8%, but was almost seven-fold higher in hypotensive patients. Major vascular complications occurred in 9.8% of our patients, similar to the literature value of 11.9%, yet they clustered in the hypotensive group (15.7% vs. 1.9%). Life-threatening/disabling bleeding was not observed in our cohort, contrasting with the 15.6% pooled rate, likely reflecting differences in patient selection, sample size but also to the accumulated procedural experience at our high-volume center. Stroke/TIA (1.6% vs. 5.7%) and AKI (2.4% vs. 7.5%) were less frequent, whereas permanent pacemaker implantation was markedly higher (31.7% vs. 13.9%), consistent with rates seen with self-expanding prostheses [12]. The prognostic impact of hypotension has been reported across cardiovascular interventions. Fefer et al. demonstrated that multiple or prolonged rapid pacing runs significantly increase the risk of acute kidney injury and mortality after TAVI [13]. Similarly, Ni et al. showed that post-induction hypotension independently predicted a composite of AKI and stroke in older TAVI patients [7]. Our findings are consistent with these observations but extend them by quantifying the mortality risk (12.9% vs. 1.9%) and identifying novel clinical predictors.

Reduced ejection fraction was strongly associated with hypotension (OR 2.87, 95% CI 1.13–7.31). Gerfer et al. showed that patients with reduced EF (≤40%) were at substantially higher perioperative risk, with greater need for mechanical ventilation, resuscitation, and circulatory support compared with those with preserved function [14]. However, the study did not specifically address perioperative hypotension. Low baseline blood pressure has also been highlighted as a prognostic marker: Butala et al. reported that preprocedural SBP < 120 mmHg predicted higher 12-month mortality after TAVI [15]; while Lindman et al. demonstrated that low blood pressure within the first month after AVR was associated with increased late mortality [16]. Our study adds granularity by demonstrating that specifically lower baseline diastolic BP is a risk factor for intra-procedural hypotension.

Notably, diabetes mellitus emerged as an independent predictor of perioperative hypotension in our cohort (OR 2.79, 95% CI 1.03–7.56). This association has not been consistently reported in earlier TAVI studies but is biologically plausible given the role of diabetic autonomic neuropathy in impairing baroreflex sensitivity, predisposing to perioperative hemodynamic instability [17,18,19]. Prior anesthesiology literature supports this link, with studies demonstrating higher rates of intraoperative hypotension in diabetic patients with autonomic dysfunction [20,21]. Our findings therefore extend the relevance of this mechanism to the TAVI population.

Procedural duration was another independent predictor of hypotension (OR 1.038 per minute). This echoes prior observations that longer procedures, often reflecting complex anatomy or vascular access challenges, accumulate more pacing episodes and anesthetic exposure, thereby amplifying hemodynamic risk. A recent consensus emphasized minimizing the total duration of hypotensive episodes during TAVI to reduce renal injury and mortality [22]. In our cohort, procedure duration did not differ significantly between patients with and without diabetes mellitus or reduced EF, and it was not correlated with baseline systolic or diastolic blood pressure. This indicates that procedural length was not simply a surrogate for these baseline risk factors but more likely reflected procedural complexity itself. Therefore, while procedure time was associated with hypotension in our cohort, causality cannot be inferred, and its role should be interpreted cautiously.

An exploratory but noteworthy observation was the significantly lower use of sugammadex in the hypotension group (11.4% vs. 32.1%, *p* = 0.005). Although sugammadex was not included in the regression model because it is administered post-procedure, this trend suggests a potential protective role. Literature on sugammadex reports more favorable hemodynamic stability compared with neostigmine [23], although rare adverse events such as bradycardia or hypotension have also been described [24,25]. Prospective evaluation is needed to clarify whether sugammadex confers clinically meaningful benefit in TAVI patients.

Our findings have several practical implications. First, patients with low EF, diabetes, and lower baseline diastolic BP should be recognized as high-risk for hypotension, warranting enhanced monitoring and anticipatory vasopressor support. Second, minimizing procedural duration and cumulative pacing burden may mitigate the risk. Finally, our data highlight the need for tailored anesthetic protocols in vulnerable subgroups and encourage further investigation into pharmacologic strategies—such as neuromuscular blockade reversal agents—that might optimize hemodynamic stability.

This study has limitations. Its retrospective and single-center nature may limit generalizability, and reliance on anesthetic records may underestimate brief hypotensive episodes. Long-term outcomes beyond hospitalization were not assessed. Nonetheless, the strengths include a well-characterized cohort, detailed intraoperative hemodynamic data, and the identification of both predictors, with robust model performance (AUC 0.844).

Taken together, our study highlights that perioperative hypotension is not merely a frequent technical complication during TAVI, but a clinically meaningful event with prognostic implications. While reduced EF and low baseline blood pressure have been recognized as markers of hemodynamic vulnerability, we uniquely demonstrate that diabetes mellitus independently predisposes patients to intra-procedural hypotension, likely reflecting underlying autonomic dysfunction. This novel finding differentiates our work from previous studies, which largely focused on ventricular function and procedural mechanics but did not systematically evaluate the role of metabolic comorbidity. Moreover, the nearly seven-fold increase in in-hospital mortality (12.9% vs. 1.9%) among hypotensive patients underscores the magnitude of risk associated with hemodynamic instability. The observation that sugammadex use was less common in patients with hypotension, although exploratory, may open a new line of inquiry into anesthetic strategies that optimize perioperative safety in this frail population.

## 5. Conclusions

Perioperative hypotension is common during TAVI and is strongly associated with increased in-hospital mortality. Diabetes mellitus, reduced EF and lower baseline diastolic blood pressure are independent predictors. Longer procedure duration was associated with hypotension in the original model, but this was not robust after bootstrap validation. These findings add to the growing body of evidence on the prognostic significance of hemodynamic instability in TAVI and uniquely identify diabetes mellitus as a novel risk factor, suggesting that targeted prevention and individualized anesthetic strategies could improve patient outcomes.

## Figures and Tables

**Table 1:** Comparison of demographic, clinical characteristics between patients with and without perioperative hypotension undergoing TAVI.

Variable	Hypotension (−) *n* = 53 (43.1%)	Hypotension (+) *n* = 70 (56.9%)	Total *n* = 123 (100%)	*p*
Sex				0.676
female	27 (50.9%)	33 (47.1%)	60 (48.8%)	
male	26 (49.1%)	37 (50.2%)	63 (51.2%)	
Age (year)	80 (51–95)	81 (57–94)	80 (51–95)	0.458
BMI (kg/m^2^)	28.6 (20.4–33.6)	27.3 (20.8–34.6)	27.4 ± 3.4	0.251
Comorbidities				
CAD	33 (63.5%)	41 (60.3%)	74 (61.7%)	0.724
Hypertension	43 (82.7%)	48 (70.6%)	91 (75.8%)	0.125
Diabetes	14 (26.9%)	29 (44.6%)	43 (36.8%)	0.049
CKD	6 (11.3%)	5 (7.2%)	11 (9.0%)	0.530 *
Stroke	2 (3.8%)	3 (4.3%)	5 (4.1%)	1.000 *
Obesity	22 (41.5%)	29 (41.4%)	51 (41.5%)	0.993
Smoking history	6 (11.3%)	8 (11.4%)	14 (11.4%)	0.985
EF (%)	55 (35–65)	50 (20–65)	55 (20–65)	0.001
Low EF	16 (31.4)	42 (61.8)	58 (48.7)	0.001
Logistic EuroScore	23.0 ± 13.4	23.5 ± 13.6	24.9 ±13.2	0.514
Medication History				
Beta-blockers	38 (71.7%)	50 (71.4%)	88 (71.5%)	0.974
ACE-İ/ARBs	37 (69.8%)	47 (68.1%)	84 (68.9%)	0.841
NOAC	29 (54.7%)	42 (60.0%)	71 (57.7%)	0.557
Warfarin	4 (7.5%)	4 (5.7%)	8 (6.5%)	0.725 *
ADP blocker	6 (11.3%)	6 (8.6%)	12 (9.8%)	0.611
Statins	16 (30.8%)	19 (27.1%)	35 (28.7%)	0.661
Loop diuretics	34 (64.2%)	40 (57.1%)	74 (60.2%)	0.432
MRAs	3 (5.8%)	7 (10.1%)	10 (8.3%)	0.513 *

ACE-I, Angiotensin-Converting Enzyme Inhibitor; ADP blocker, Adenosine Diphosphate Receptor Inhibitor; ARBs, Angiotensin II Receptor Blockers; BMI, Body Mass Index; CAD, Coronary Artery Disease; CKD, Chronic Kidney Disease; EF, Ejection Fraction; Low EF, Ejection fraction < 50%; MRAs, Mineralocorticoid Receptor Antagonists; NOAC, Non-Vitamin K Antagonist Oral Anticoagulant. * Fisher’s Exact Test.

**Table 2:** Baseline hemodynamics, procedural characteristics, and Valve Academic Research Consortium-2 (VARC-2) clinical outcomes in patients with and without perioperative hypotension.

	Hypotension (−)	Hypotension (+)	Total	*p*
Preoperative SBP (mmHg)	150 (108–200)	129 (93–220)	141.2 ± 28.9	<0.001
Preoperative DBP (mmHg)	66.3 ± 8.8	58.3 ± 12.5	60.0 ± 12.1	<0.001
Preoperative HR (/dk)	76 ± 8.3	76 ± 8.4	78 (52–110)	0.826
General anesthesia	20 (37.7%)	18 (24.6%)	38 (30.9%)	0.109
Mean NIRS (%)	63.5 (29–73)	64 (35–92)	64 (29–92)	0.339
Body Temperature (°C)	37.0 (36.1–38.0)	37.0 (35.9–37.3)	37.0 (35.9–38.0)	0.262
Procedure time (min)	60 (35–120)	70 (50–120)	65 (35–120)	0.005
Total anesthetic time (min)	75 (45–140)	80 (55–130)	80 (45–140)	0.032
**OUTCOME (VARC-2)**				
Device success	1 (1.9%)	9 (12.9%)	10 (8.1%)	0.027
Life-threatening/disabling bleeding	0 (0%)	0 (0%)	0 (0.0%)	1.000
Bleeding (mL) *	200 (100–350)	250 (150–350)	200 (100–350)	0.037
Major vascular complication	1 (1.9%)	11 (15.7%)	12 (9.8%)	0.010
Stage 1–3 AKI	0 (0%)	3 (4.3%)	3 (2.4%)	0.258
30-day stroke/TIA	0 (0%)	2 (2.9%)	2 (1.6%)	0.322
Myocardial infarction	1 (0%)	1 (0%)	2 (1.6%)	0.678
Arrythmia *	19 (35.8%)	25 (35.7%)	44 (35.8%)	0.988
New permanent pacemaker	15 (28.3%)	24 (34.3%)	39 (31.7%)	0.306
Length of ICU stay (days) *	1 (1–2)	1 (1–31)	1 (1–31)	0.032
30-day or in-hospital mortality	1 (1.9%)	9 (12.9%)	10 (8.1%)	0.027

AKI, acute kidney injury; DBP, Diastolic Blood Pressure; HR, Heart Rate; ICU, Intensive Care Unit; NIRS, Near-Infrared Spectroscopy; SBP, Systolic Blood Pressure; TIA, transient ischemic attack; VARC-2, Valve Academic Research Consortium-2 criteria. * Not part of VARC-2, added for descriptive purposes.

**Table 3:** Comparison of preoperative laboratory parameters between patients with and without perioperative hypotension undergoing TAVI.

Laboratory Data	Hypotension (−)	Hypotension (+)	Total	*p*
Albumin (g/dL)	3.6 ± 0.3	3.6 ± 0.4	3.6 (2.0–4.5)	0.547
Total cholesterol (mg/dL)	160 ± 41.4	158 ± 48.8	158 (39–309)	0.691
HDL (mg/dL)	38 (20–69)	38.5 (21–125)	39 (20–125)	0.941
LDL (mg/dL)	101 (39–171)	94.5 (19–224)	100.5 (19–224)	0.307
TG (mg/dL)	87 (37–320)	116 (31–300)	106 (37–320)	0.016
Hgb (g/dL)	10.9 ± 1.39	10.9 ± 1.68	11.2 ±1.71	0.844
Hct (%)	33.4 ± 3.79	33.6 ± 4.82	34.3 ± 4.94	0.834
Plt (10^9^/L)	217 (123–393)	204 (111–622)	214 (111–622)	0.994
ALT (U/L)	11 (6–142)	16.5 (6–362)	15 (6–362)	0.064
AST (U/L)	17 (4–84)	21.5 (11–114)	19 (4–114)	0.147
GGT (U/L)	17 (3–175)	23.5 (9–360)	21.5 (3–360)	0.336
Total bilirubine (mg/dL)	0.59 (0.27–3.41)	0.67 (0.17–7.4)	0.65 (0.17–7.4)	0.095

ALT, Alanine Aminotransferase; AST, Aspartate Aminotransferase; GGT, Gamma-Glutamyl Transferase; Hct, Hematocrit; HDL, High-Density Lipoprotein; Hgb, Hemoglobin; LDL, Low-Density Lipoprotein; Plt, Platelet Count; TG, Triglyceride.

**Table 4:** Comparison of perioperative anesthetic drug use between patients with and without perioperative hypotension undergoing TAVI.

Anesthetics	Hypotension (−)	Hypotension (+)	Total	*p*
Midazolam				0.050
1–2 mg	40 (75.5%)	62 (88.6%)	102 (82.9%)	
3–4 mg	13 (24.5%)	8 (11.4%)	21 (17.1%)	
Dexmedetomidine (mcg)	1.18 (0.6–2.0)	1.16 (0.6–2.0)	1.17 (0.6–2.0)	0.312
Remifentanil	15 (28.3%)	18 (25.7%)	33 (26.8%)	0.748
Rocuronium	19 (35.8%)	16 (22.9%)	35 (28.5%)	0.114
Sugammadex	17 (32.1%)	8 (11.4%)	25 (20.3%)	0.005
Propofol	53 (100%)	70 (100%)	123 (100%)	N/A

**Table 5:** Results of univariate and multivariate logistic regression analyses for predictors of perioperative hypotension in patients undergoing TAVI.

	Univariate Analysis		Multivariate Analysis		Bootstrap Analysis	
	OR (95% CI)	*p*	OR (95% CI)	*p*	OR (95% CI)	*p*
Hypertension	0.502 (0.207–1.221)	0.129				
Diabetes	2.187 (0.998–4.789)	0.050	2.787 (1.027–7.561)	0.044	2.63 (1.09–7.99)	0.036
Low EF	3.534 (1.640–7.613)	0.001	2.870 (1.126–7.313)	0.027	3.03 (1.19–7.87)	0.016
Preoperative SBP (mmHg)	0.977 (0.963–0.991)	0.001				
Preoperative DBP (mmHg)	0.934 (0.900–0.968)	<0.001	0.935 (0.893–0.978)	0.004	0.94 (0.87–0.97)	0.002
Vascular Injury	9.695 (1.210–77.664)	0.032				
Procedure time (min)	1.029 (1.001–1.058)	0.045	1.038 (1.001–1.076)	0.044	1.03 (0.99–1.09)	0.114
TG (mg/dL)	1.007 (0.999–1.015)	0.094				
ALT (U/L)	1.006 (0.992–1.021)	0.371				
Total bilirubine (mg/dL)	1.257 (0.724–2.183)	0.417				
Midazolam (1–2 mg = 1, 3–4 mg = 0)	2.519 (0.958–6.620)	0.061				

## Data Availability

The data presented in this study are available on reasonable request from the corresponding author. The data are not publicly available due to privacy restrictions.
